# Post-transplantation management of hyperparathyroidism and its association with kidney graft survival and fibrosis

**DOI:** 10.1007/s10157-025-02723-7

**Published:** 2025-07-04

**Authors:** Manabu Okada, Tetsuhiko Sato, Tomoki Himeno, Yuki Hasegawa, Yuki Shimamoto, Kenta Futamura, Takahisa Hiramitsu, Norihiko Goto, Shunji Narumi, Asami Takeda, Toshihiro Ichimori, Yoshihiko Watarai

**Affiliations:** 1 Department of Transplant Surgery and Transplant Nephrology, Japanese Red Cross Aichi Medical Center Nagoya Daini Hospital, Nagoya, Japan; 2 Department of Diabetes and Endocrinology, Japanese Red Cross Aichi Medical Center Nagoya Daini Hospital, Nagoya, Japan; 3 Department of Nephrology, Masuko Memorial Hospital, Nagoya, Japan

**Keywords:** Kidney transplantation, Hyperparathyroidism, Parathyroidectomy, Calcimimetics, Interstitial fibrosis and tubular atrophy

## Abstract

**Background:**

Hyperparathyroidism (HPT) is a potential risk factor for graft loss after kidney transplantation (KTx). However, the effects of HPT management on graft outcomes remain unclear. This retrospective study aimed to investigate the impact of HPT status and its management on graft outcomes.

**Methods:**

Patients who underwent KTx were categorized based on their HPT status and treatment at 1-year post-KTx into four groups: normal (no HPT), normocalcemic HPT, hypercalcemic HPT, or intervention (parathyroidectomy or calcimimetics within 1 year after KTx). Patients treated for HPT beyond the first year post-KTx were censored. The primary outcome was death-censored graft survival and the secondary outcome was the progression of interstitial fibrosis and tubular atrophy (IFTA) at 1-year post KTx.

**Results:**

Among 1264 patients, the 10-year death-censored graft-survival rate was lowest in the hypercalcemic HPT group (79.7%), whereas the intervention group had a survival rate of 100.0%. In the multivariate Cox regression analysis, hypercalcemic HPT was associated with an increased risk of graft loss (fully adjusted hazard ratio [HR] = 4.25, *P* = 0.001, compared to the normal group). Contrarily, the intervention group did not show an increased risk of graft loss (fully adjusted HR = 0.28, *P* = 0.239). Additionally, hypercalcemia during the first year after KTx was significantly associated with IFTA progression (fully adjusted odds ratio = 1.91, *P* = 0.038).

**Conclusion:**

Hypercalcemic HPT was associated with inferior graft survival and IFTA progression. Proactive management of HPT may reduce the risk of graft loss and mitigate IFTA progression.

**Supplementary Information:**

The online version contains supplementary material available at 10.1007/s10157-025-02723-7.

## Introduction

Hyperparathyroidism (HPT) is a common complication associated with chronic kidney disease (CKD), leading to cardiovascular and bone diseases and mortality in patients with CKD [1]. After a successful kidney transplantation (KTx), HPT resolves to some extent by improving kidney function [2]. However, HPT often persists even after KTx and is associated with unfavorable kidney grafts and patient outcomes [3, 4]. Pihlstrøm et al. demonstrated that HPT was an independent risk factor for kidney graft loss and all-cause mortality in their retrospective study of 1,820 patients who underwent KTx [3]. Lou et al. also reported that persistent HPT was associated with poorer kidney graft survival in their study of 1,609 KTx patients [4]. Additionally, Araujo et al. demonstrated the association between HPT and the long-term risk of kidney graft failure in a retrospective study of 911 KTx patients [5].

Hypercalcemia is internationally recognized as one of the most common therapeutic indications for post-KTx HPT. The American Association of Endocrine Surgeons guidelines recommend parathyroidectomy (PTx) for patients with hypercalcemic HPT after KTx [6]. Similarly, the guidelines of the Japanese Society for Dialysis Therapy suggest intervention for persistent hypercalcemic HPT 1 year after KTx [7]. However, the efficacy of interventions for HPT in improving graft outcomes and the underlying mechanisms by which HPT adversely impacts graft prognosis remain poorly understood. Some reports suggest that HPT may contribute to the progression of pathological fibrosis in kidney grafts [8, 9].

Therefore, we hypothesized that hypercalcemic HPT plays a role in the progression of interstitial fibrosis and tubular atrophy (IFTA), a pathological condition characterized by scarring and structural deterioration in the kidney, often leading to chronic graft dysfunction [10]. Hence, this retrospective study aimed to evaluate the impact of HPT status and its management on kidney graft survival and the progression of IFTA.

## Materials and methods

### Data source

This study included consecutive patients who underwent KTx at the Japanese Red Cross Aichi Medical Center Nagoya Daini Hospital between January 2008 and June 2022. Data collection was completed on July 16, 2024.

### Participants and study design

The exclusion criteria were as follows: patients (1) who underwent PTx before KTx, (2) under 18 years of age at the time of KTx, (3) with missing data, and (4) with end-stage kidney disease with an estimated glomerular filtration rate (eGFR) < 15 mL/min/1.73 m^2^ within the first year after KTx. To avoid survivor treatment selection bias, patients who received treatment for HPT beyond the first year after KTx were censored at treatment initiation. Data on age; sex; body mass index (BMI); original disease; dialysis duration; donor type; serum calcium (Ca), phosphorus (P), hemoglobin (HGB), low-density lipoprotein cholesterol (LDL-C), uric acid (UA), calcineurin inhibitors (CNI), and intact parathyroid hormone (PTH) levels; eGFR; urinary protein; mean blood pressure; ABO blood type incompatibility; positivity for preformed donor-specific human leukocyte antigen antibodies (DSA); PTx and calcimimetic treatment histories; and graft survival were collected. In addition, IFTA was assessed by reviewing reports of protocol biopsies performed at 1-h and 1-year post-KTx. Information on rejection within 1 year after KTx and tubulointerstitial calcification was also collected from the reports of protocol and indication biopsies. The rejection and IFTA were graded by an experienced transplant pathologist according to the Banff classification system.

Hypercalcemia was defined as total serum Ca levels ≥ 10.5 mg/dL [7]. Hyperparathyroidism was defined as intact PTH levels > 80 pg/mL [7, 11]. Patients who met the inclusion criteria were divided into four groups based on the status and management of HPT at 1-year post-KTx: the normal group, comprising patients without HPT; normocalcemic HPT group, comprising patients with normocalcemic HPT; hypercalcemic HPT group, comprising patients with hypercalcemic HPT; and intervention group, comprising patients who underwent PTx or initiated treatment with calcimimetics for hypercalcemic HPT within the first year after KTx. The primary outcome was death-censored graft-survival rate. The secondary outcome was progression of IFTA. The risk factors for death-censored graft loss and IFTA progression were analyzed using multivariate analysis. All patients were followed up at our institution for 1 year after KTx. Follow-up data on HPT treatment, graft loss, and death were obtained from the patients’ new care institutions after transfer.

### Measurements

Serum Ca and P levels were measured using standard methods. Intact PTH levels were measured using the following second-generation immunoassays: an electrochemical luminescence immunoassay (SRL, Tokyo, Japan, www.srl-group.co.jp; reference range: 10–65 pg/mL) and an enzyme immunoassay (Tosoh, Tokyo, Japan, www.tosoh.co.jp; reference range: 9–80 pg/mL). For serum albumin levels < 4.0 g/dL, all serum Ca levels were corrected [12]. The eGFR was evaluated using the creatinine equation provided by the Japanese Society of Nephrology [13]. Post-KTx blood sample analyses were performed monthly for 1 year after KTx.

### Management of HPT

At our institution, hypercalcemia is the primary treatment criterion for HPT after KTx. Before 2008, PTx was the first-line treatment; however, since then, calcimimetics have often been used first, based on physician and patient preferences. Normocalcemic HPT is not treated but only monitored.

### Immunosuppression

Immunosuppressive regimens included the administration of CNI (cyclosporine or tacrolimus), mycophenolic acids, mizoribine, everolimus, and glucocorticoids. Basiliximab was used for induction therapy. In addition, rituximab administration or splenectomy was used as induction therapy in anti-donor antibody-positive patients before KTx, except in those with low antibody titers.

### Statistical analysis

Pearson’s chi-squared test was used to analyze nominal variables and Kruskal–Wallis test was used for continuous variables. All results are presented as median (interquartile range [IQR]) because of their non-normal distribution, as confirmed by Shapiro–Wilk normality test and histogram. Statistical. significance was set at *P* < 0.05.

Kaplan–Meier survival curves and log-rank test were used to estimate and compare the graft survival rates. To investigate the association of HPT management with death-censored graft loss, univariate and multivariate Cox regression analyses were performed. Model 1 was adjusted for recipient age and sex. In Model 2, eGFR and proteinuria were added due to their potential influence on graft prognosis. Model 3 was additionally adjusted for nonimmunologic transplant-related factors (BMI, dialysis duration, and donor age and type). Model 4 was adjusted for immunologic factors (rejection events and preformed DSA) and factors included in Model 3. Model 5 was adjusted for other potential confounders related to graft outcomes (P, HGB, UA, and mean blood pressure at 1-year post-KTx) in addition to the factors included in Model 4. For sensitivity analysis, stratified HRs of death-censored graft loss for the hypercalcemic HPT group, with reference to the normal group, were also analyzed within subgroups classified by sex, dialysis duration, BMI, eGFR, donor type, UA, and presence of IFTA at 1-year post-KTx due to imbalances between the groups.

Logistic regression analysis was performed to assess the impact of hypercalcemia on the new onset or progression of IFTA at 1-year post-KTx. To evaluate the cumulative effects of exposure to various parameters over the first year on IFTA progression, we used the average values of serum Ca, P, HGB, LDL, UA, intact PTH, and CNI levels during this period. Hypercalcemia was analyzed both as a categorical variable (absent vs. mean serum Ca level ≥ 10.5 mg/dL) and as a continuous variable (per 1 mg/dL increase in serum Ca). Model 1 was adjusted for recipient age and sex. In Model 2, eGFR and proteinuria were added as factors associated with graft damage. Model 3 was further adjusted for donor age and donor type due to their influence on the quality of the kidney graft. Model 4 was adjusted for rejection events, preformed DSA, and the factors included in Model 3 as immunological factors that may damage the kidney graft. Model 5 was adjusted for other potential risk factors related to IFTA progression (BMI, dialysis duration, diabetic kidney disease, mean blood pressure, and serum P, HGB, LDL-C, UA, intact PTH, CNI levels) and the factors included in Model 4.

In these analyses, CNI trough levels were categorized into three tertiles: low, medium, and high. Proteinuria was also categorized into three groups based on severity: normal (< 0.15 g/g creatinine), mild (0.15–0.49 g/g creatinine), and severe (≥ 0.50 g/g creatinine) [14].

Easy R (EZR) version 1.61 (The R Foundation for Statistical Computing) was used for statistical analyses [15].

## Results

### Participant characteristics

**Fig. 1:**
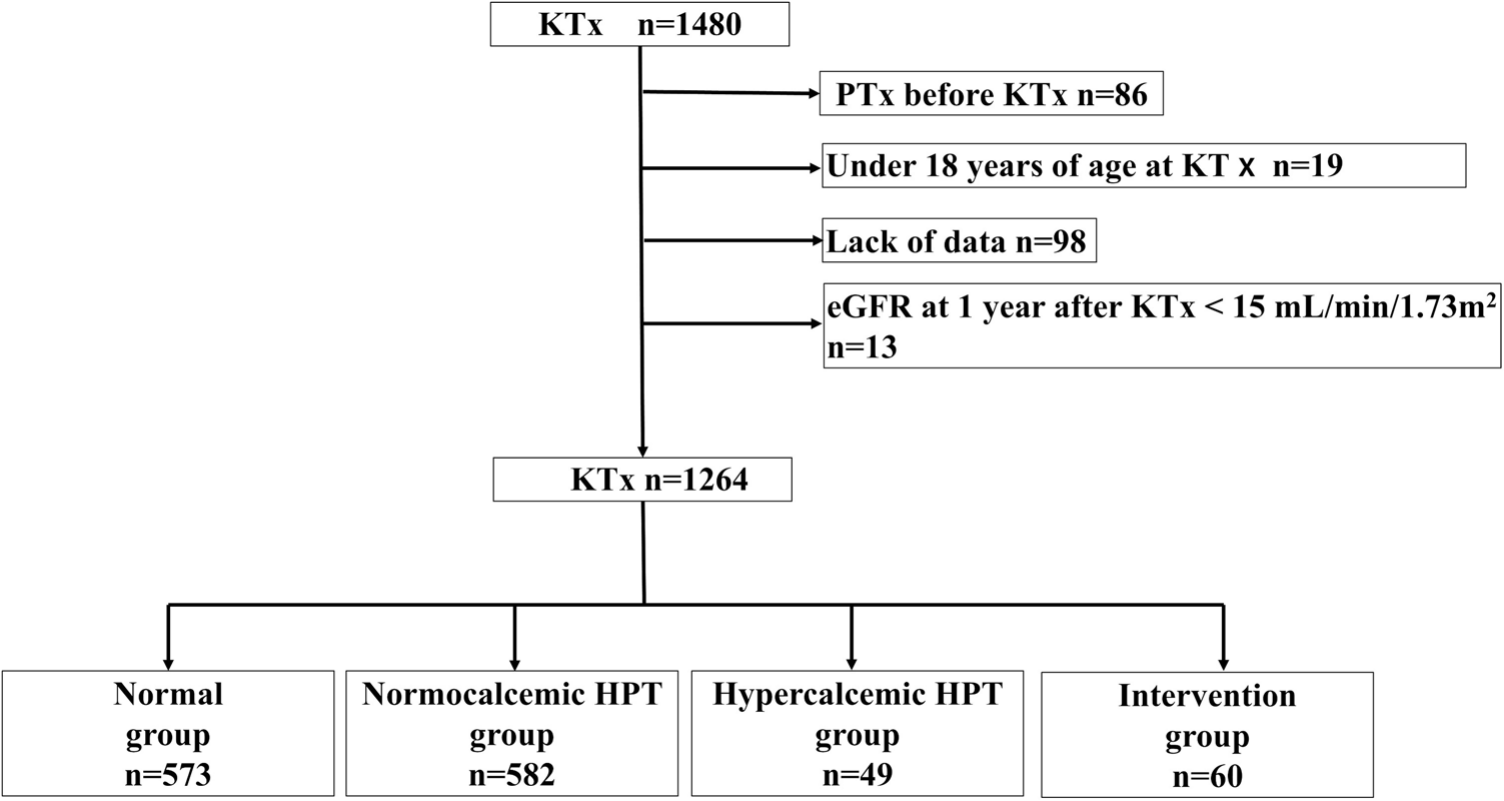
Participant selection flowchart. *eGFR* *estimated glomerular filtration rate*, *KTx* *kidney transplantation*, *PTx* *parathyroidectomy*, *HPT* *hyperparathyroidism*

A total of 1264 patients met the inclusion criteria (median observation period, 96 months [IQR: 59–142 months]; Fig. 1). Of the 1264 patients, 573, 582, 49, and 60 were assigned to the normal, normocalcemic HPT, hypercalcemic HPT, and intervention groups, respectively (Fig. 1). Significant differences were observed in sex; dialysis duration; BMI; original disease; donor age; donor type; serum Ca, P, intact PTH, and UA levels; eGFR; and urinary protein among the four groups (Table 1). However, recipient age, preformed DSA, ABO blood type incompatibility, HGB, LDL-C, CNI trough levels, mean blood pressure, biopsy-proven rejection within 1 year after KTx, and follow-up period did not differ between the groups (Table 1). In the intervention group, 33 patients underwent PTx, 27 started calcimimetics use within the first year after KTx, and 15 underwent PTx 1 year after initial calcimimetic treatment. Hypercalcemia resolved in all patients following treatment. In the hypercalcemic HPT group, 15 patients underwent PTx or initiated calcimimetic treatment for hypercalcemia more than 1 year after KTx, whereas in the normocalcemic HPT group, one patient underwent PTx in conjunction with thyroid tumor resection during the same period.

**Table 1:** Patient characteristics and clinical outcomes

	Normal *N* = 573	Normocalcemic HPT *N *= 582	Hypercalcemic HPT *N* = 49	Intervention *N* = 60	*P*-value
Baseline characteristics					
Recipient age (years)	50 [38–61]	47 [39–61]	53 [44–61]	54 [45–62]	0.106
Recipient sex (male, %)	355 (62.0)	390 (67.0)	31 (63.3)	26 (43.3)*	0.003
Dialysis duration (months)	0 [0–10]	4 [0–24]*	87 [13–120]*	126 [72–175]*	< 0.001
Body mass index (kg/m^2^)	21.5 [19.5–24.1]	22.1 [19.8–24.9]	22.6 [19.9–24.9]	20.7 [18.9–22.8]	0.002
Cause of ESKD (%)					0.034
Glomerular disease	212 (37.0)	202 (34.7)	18 (36.7)	27 (45.0)	
Diabetic kidney disease	126 (22.0)	114 (19.6)	14 (28.6)	8 (13.3)	
Polycystic kidney disease	50 (8.7)	47 (8.1)	6 (12.2)	6 (10.0)	
Hypertensive kidney disease	33 (5.8)	50 (8.6)	2 (4.1)	0 (0.0)	
Others	52 (9.1)	49 (8.4)	7 (14.3)	9 (15.0)	
Unknown	100 (17.5)	120 (20.6)	2 (4.1)	10 (16.7)	
Donor age (years)	60 [51–66]	60 [53–67]	58 [47–62]	57 [48–64]	0.018
Donor sex (male, %)	222 (38.7)	197 (33.8)	18 (36.7)	33 (55.0)	0.009
Donor type (deceased, %)	13 (2.3)	25 (4.3)	8 (16.3) *	13 (21.7)*	< 0.001
Preformed DSA (%)	47 (8.2)	31 (5.3)	0 (0.0)	1 (1.7)	0.089
ABO blood type incompatible KTx (%)	189 (33.0)	179 (30.8)	20 (40.8)	24 (40.0)	0.268
Lab data 1 year post-KTx					
Calcium (mg/dL)	9.6 [9.4–9.8]	9.5 [9.3–9.8]	10.8 [10.6–11.1]*	9.7 [9.1–10.4]	< 0.001
Phosphorus (mg/dL)	3.3 [3.0–3.7]	3.2 [2.8–3.5]*	2.7 [2.5–3.0]*	3.3 [2.8–3.7]	< 0.001
Intact PTH (pg/mL)	61.0 [49.0–70.9]	107.0 [92.5–136.8]*	148.0 [119.0–206.0]*	64.5 [25.5–140.8]	< 0.001
Hemoglobin (g/dL)	12.6 [11.6–13.8]	12.8 [11.7–14.0]	12.8 [11.7–13.6]	12.2 [11.1–13.3]	0.082
LDL-C (mg/dL)	99.0 [85.0–112.0]	99.0 [84.0–114.0]	96.0 [78.0–107.0]	101.0 [89.0–113.5]	0.362
Uric acid (mg/dL)	6.2 [5.3–7.2]	6.3 [5.4–7.3]	7.0 [6.0–7.6] *	6.8 [5.8–7.8]	0.004
eGFR (mL/min/1.73m^2^)	45.0 [38.2–52.4]	42.7 [35.2–50.3] *	43.9 [36.2–51.9]	44.0 [35.0–50.8]	0.001
Urinary protein (g/g creatinine)	0.06 [0.02–0.13]	0.09 [0.04–0.19] *	0.04 [0.00–0.10]	0.04 [0.00–0.13]	< 0.001
Tacrolimus trough level (ng/mL)	4.5 [3.8–5.4]	4.6 [3.9–5.6]	5.0 [3.9–5.7]	4.9 [4.2–5.8]	0.119
Cyclosporine trough level (ng/mL)	80.0 [64.0–104.0]	73.0 [53.0–101.0]	76.0 [68.8–109.5]	79.0 [67.5–102.3]	0.304
Mean blood pressure (mmHg)	90.0 [84.7–95.0]	90.3 [84.7–96.0]	86.7 [83.7–94.3]	87.3 [82.6–92.8]	0.154
Clinical outcomes					
Follow-up period (months)	94 [61–143]	98 [58–141]	83 [40–128]	102 [63–140]	0.178
BPR within 1 year after KTx (%)	38 (4.9)	36 (6.2)	3 (6.1)	1 (1.7)	0.441
Graft loss (%)	36 (6.3)	51 (8.8)	9 (18.4)*	1 (1.7)	0.004
Death (%)	26 (4.5)	48 (8.2)	6 (12.2)	5 (8.3)	0.027

*BPR* biopsy-proven rejection, *DSA* donor-specific human leukocyte antigen antibody, *eGFR* estimated glomerular filtration rate, *ESKD* end-stage kidney disease, *HPT* hyperparathyroidism, *KTx* kidney transplantation, *LDL-C* low-density lipoprotein cholesterol, *PTH* parathyroid hormone

### Graft survival

**Fig. 2:**
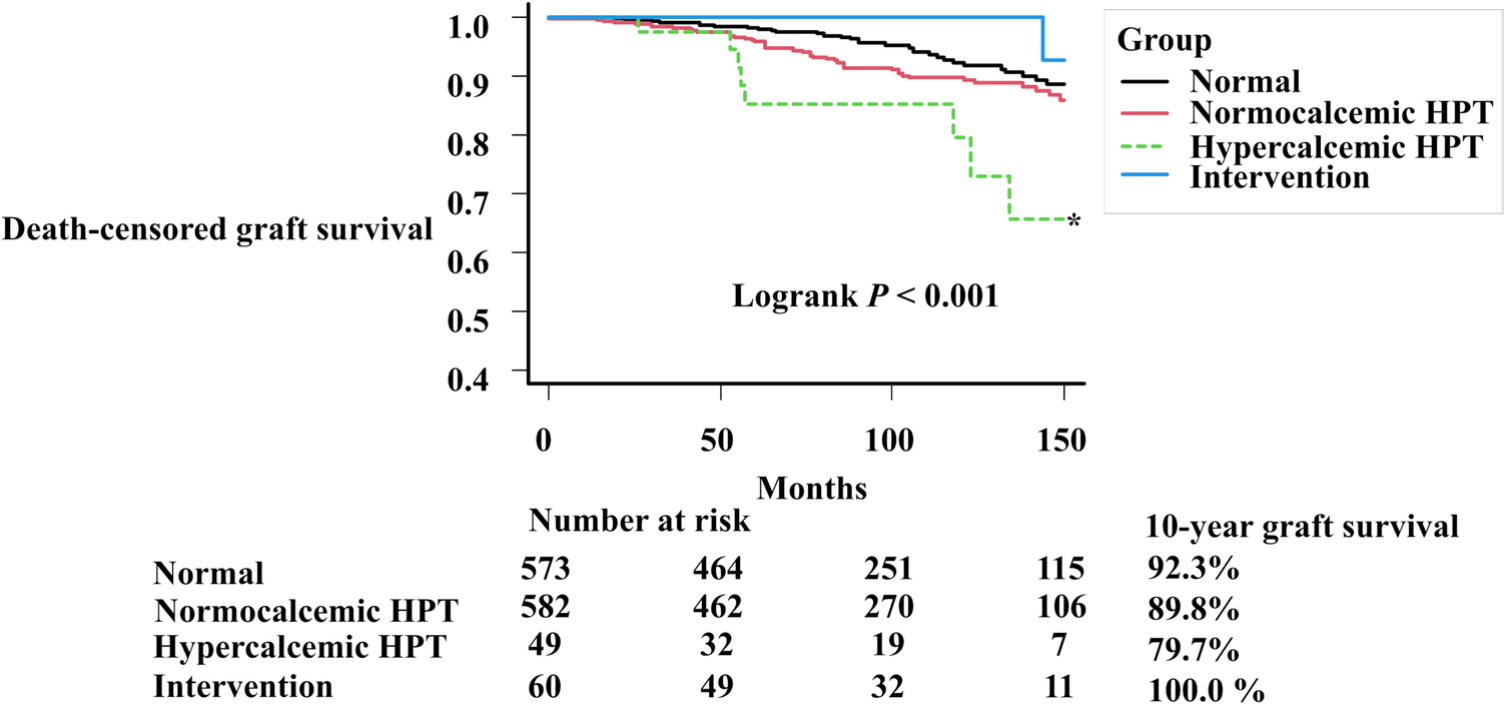
Death-censored graft survival curves according to the status of hyperparathyroidism. *HPT* *hyperparathyroidism*. * *P* < 0.05, adjusted by Bonferroni correction (vs. the normal group)

Graft loss occurred in 97 patients (Table 1). Among the four groups, the hypercalcemic HPT group showed the lowest death-censored graft survival, whereas the normocalcemic HPT and intervention groups did not differ significantly from the normal group, with 10-year survival rates of 92.8%, 89.8%, 79.7%, and 100.0%, respectively (*P* < 0.001; Fig. 2).

### Association between HPT management status and death-censored graft loss

Univariate Cox proportional hazards analysis revealed that hypercalcemic HPT was significantly associated with death-censored graft loss (*P* < 0.001; hazard ratio [HR], 3.77; 95% confidence interval [CI], 1.81–7.83; Table 2). This association remained significant after adjusting for potential confounders (*P* = 0.001; fully adjusted HR, 4.25; 95% CI 1.75–10.35; Table 2, Online Resource 1, 2). On the other hand, compared to the normal group, the normocalcemic HPT group had a higher HR, while the intervention group had a lower HR; however, neither of these differences was statistically significant.

**Table 2:** Cox regression analysis of HPT management and the risk of death-censored graft loss

Model	HPT management status	HR [95% CI]	*P*-value
Crude	Normal	Reference	–
	Normocalcemic HPT	1.44 [0.94–2.21]	0.092
	Hypercalcemic HPT	3.77 [1.81–7.83]	< 0.001
	Intervention	0.25 [0.03–1.84]	0.174
Model 1	Normal	Reference	–
	Normocalcemic HPT	1.43 [0.94–2.20]	0.098
	Hypercalcemic HPT	3.64 [1.75–7.59]	< 0.001
	Intervention	0.28 [0.04–2.06]	0.212
Model 2	Normal	Reference	–
	Normocalcemic HPT	1.23 [0.80–1.90]	0.350
	Hypercalcemic HPT	3.37 [1.58–7.18]	0.002
	Intervention	0.22 [0.03–1.65]	0.280
Model 3	Normal	Reference	–
	Normocalcemic HPT	1.24 [0.79–1.93]	0.350
	Hypercalcemic HPT	3.30 [1.43–7.61]	0.005
	Intervention	0.25 [0.03–1.99]	0.190
Model 4	Normal	Reference	–
	Normocalcemic HPT	1.32 [0.84–2.06]	0.224
	Hypercalcemic HPT	3.79 [1.63–8.81]	0.002
	Intervention	0.29 [0.03–2.42]	0.253
Model 5	Normal	Reference	–
	Normocalcemic HPT	1.37 [0.87–2.15]	0.171
	Hypercalcemic HPT	4.25 [1.75–10.35]	0.001
	Intervention	0.28 [0.03–2.34]	0.239

Model 1 was adjusted for recipient age and sex. Model 2: model 1 plus eGFR and urinary protein at 1 year post-KTx. Model 3: model 2 plus body mass index, dialysis duration, donor age and, donor type. Model 4: model 3 plus rejection events within 1 year after KTx, and preformed DSA. Model 5: model 4 plus mean blood pressure, serum phosphate, hemoglobin, and uric acid levels at 1 year post KTx

*95% CI* *95% confidence interval*; *DSA* *donor-specific human leukocyte antigen antibody*, *eGFR* *estimated glomerular filtration rate*, *HPT* *hyperparathyroidism*, *HR* *hazard ratio*, *KTx kidney transplantation*

**Fig. 3:**
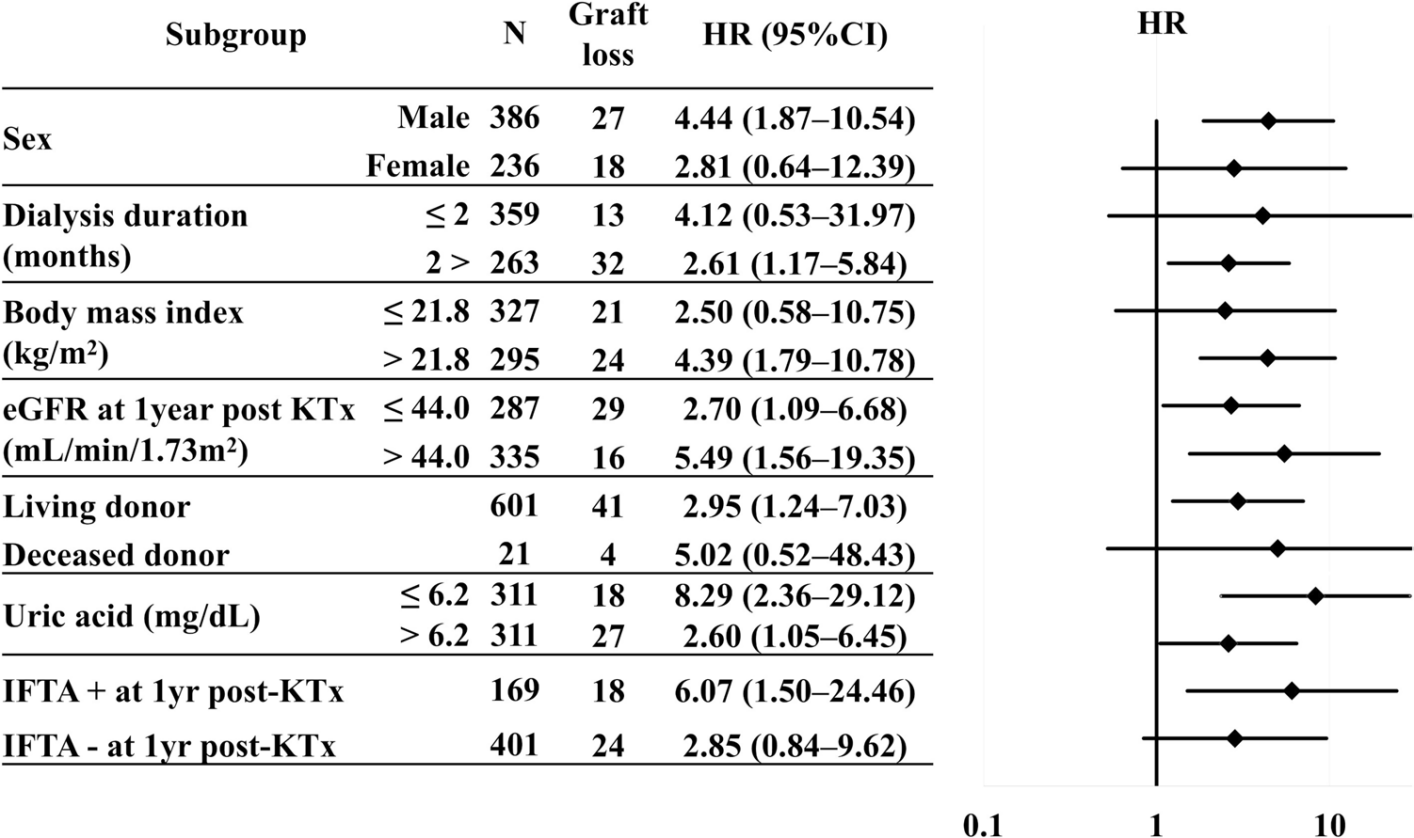
Stratified hazard ratios for death-censored graft loss in the hypercalcemic HPT group, with reference to the normal group. Dialysis duration, body mass index, eGFR, and uric acid were stratified by the median value of the entire cohort. The HRs were not adjusted for other confounders. *95% CI* 95% confidence interval, *eGFR* estimated glomerular filtration rate, *HR* hazard ratio, *HPT* hyperparathyroidism, *IFTA* interstitial fibrosis and tubular atrophy, *KTx* kidney transplantation

Stratified analysis showed that in most categories, hypercalcemic HPT tended to increase the risk of graft loss, although this was not necessarily statistically significant in all strata because of the reduced number of events in each group. When stratified by the presence of IFTA at 1-year post-KTx, the impact of hypercalcemic HPT on graft loss was greater in the IFTA-positive group than in the IFTA-negative group (Fig. 3).

### IFTA in protocol graft biopsy

Protocol graft biopsies were performed in 1,257 and 1,129 patients at 1-h and 1-year post-KTx, respectively. New-onset or progression of IFTA was observed in 289 patients (121 (23.0%), 134 (26.6%), 15 (33.3%), and 19 (36.5%) in the normal, normocalcemic HPT, hypercalcemic HPT, and intervention groups, respectively, *P* = 0.079) (Table 3). Univariate logistic regression analysis identified that hypercalcemia during the first year after KTx was significantly associated with IFTA progression (*P* = 0.019; odds ratio [OR], 1.82; 95% CI, 1.10–2.99; Table 4). Multivariate logistic regression further confirmed a significant association between hypercalcemia during the first year after KTx and IFTA progression (*P* = 0.038; fully adjusted OR 1.91; 95% CI, 1.04–3.51; Table 4, Online Resource 3, 4).

**Table 3:** IFTA and tubulointerstitial calcification in kidney graft

	Normal	Normocalcemic HPT	Hypercalcemic HPT	Intervention	*P*-value
Protocol biopsy one hour post KTx	*N* = 572	*N* = 577	*N* = 49	*N* = 59	
Chronic interstitial score (%)					0.029
0	517 (90.4)	526 (91.2)	45 (91.8)	49 (83.1)*	
1	54 (9.4)	48 (8.3)	4 (8.2)	8 (13.6)*	
2	1 (0.2)	3 (0.5)	0 (0.0)	2 (3.4)*	
Chronic tubular score					
0	372 (65.0)	391 (67.8)	38 (77.6)	39 (66.1)	0.045
1	198 (34.6)	183 (31.7)	11 (22.4)	18 (30.5)	
2	2 (0.3)	3 (0.5)	0 (0.0)	2 (3.4)	
IFTA (%)					0.044
I	53 (9.3)	45 (7.8)	4 (8.2)	6 (10.2)*	
≥ II	1 (0.2)	3 (0.5)	0 (0.0)	2 (3.4)*	
Tubulointerstitial calcification (%)	3 (0.5)	3 (0.5)	2 (4.1)	0 (0.0)	0.020
Protocol biopsy 1 year post KTx	*N* = 526	*N* = 505	*N* = 45	*N* = 53	
Chronic interstitial score (%)					0.176
0	372 (70.7)	344 (68.1)	29 (64.4)	27 (50.9)	
1	126 (24.0)	120 (23.8)	13 (28.9)	20 (37.7)	
2	25 (4.8)	34 (6.7)	3 (6.7)	5 (9.4)	
3	3 (0.6)	7 (1.4)	0 (0.0)	1 (1.9)	
Chronic tubular score					0.233
0	189 (35.9)	186 (36.8)	19 (42.2)	11 (20.8)	
1	307 (58.4)	278 (55.0)	23 (51.1)	35 (66.0)	
2	27 (5.1)	34 (6.7)	3 (6.7)	6 (11.3)	
3	3 (0.6)	7 (1.4)	0 (0.0)	1 (1.9)	
IFTA (%)					0.093
I	125 (23.8)	118 (23.5)	13 (28.9)	20 (37.7)	
≥ II	28 (5.3)	41 (8.2)	3 (6.7)	6 (11.3)	
Tubulointerstitial calcification (%)	12 (2.3)	27 (5.3)	3 (6.7)	6 (11.3)*	0.003
New onset or progression of IFTA	121 (23.0)	134 (26.6)	15 (33.3)	19 (36.5)	0.079

**Table 4:** Logistic regression analysis of serum calcium levels during the first year after KTx and their association with IFTA progression

	Hypercalcemia, OR [95% CI]			Average serum calcium level per 1 mg/dL	
Model	Absent	Present	*P*-value	OR [95% CI]	*P*-value
Crude	Reference	1.82 [1.10–2.99]	0.019	1.36 [1.02–1.81]	0.036
Model 1	Reference	1.82 [1.10–3.00]	0.020	1.34 [1.01–1.79]	0.046
Model 2	Reference	1.90 [1.14–3.17]	0.014	1.46 [1.08–1.96]	0.013
Model 3	Reference	1.84 [1.10–3.09]	0.021	1.42 [1.05–1.93]	0.022
Model 4	Reference	1.85 [1.10–3.10]	0.020	1.44 [1.07–1.96]	0.018
Model 5	Reference	1.91 [1.04–3.51]	0.038	1.51 [1.04–2.19]	0.031

Model 1 was adjusted for recipient age and sex. Model 2: model 1 plus eGFR and urinary protein at 1 year post-KTx. Model 3: model 2 plus donor age and, donor type. Model 4: model 3 plus rejection events within 1 year after KTx, and preformed DSA. Model 5: model 4 plus body mass index, dialysis duration, serum phosphorus, hemoglobin, LDL-C, uric acid, intact PTH, CNI levels, diabetic kidney disease, and mean blood pressure

The average values of serum calcium, phosphorus, hemoglobin, LDL-C, uric acid, intact PTH, and CNI levels during the first year after KTx were used Hypercalcemia was defined as an average serum calcium level of ≥ 10.5 mg/dL during the first year after KTx

*95% CI*, 95% confidence interval, *CNI* calcineurin inhibitor, *DSA* donor-specific human leukocyte antigen antibody, *eGFR* estimated glomerular filtration rate, *IFTA* interstitial fibrosis and tubular atrophy, *KTx* kidney transplantation, *LDL-C* low-density lipoprotein cholesterol, *OR* odds ratio, *PTH* parathyroid hormone

In the intervention group, patients with IFTA progression had a significantly longer interval between KTx and treatment intervention than those without progression (Table 5).

**Table 5:** Patient backgrounds with and without IFTA progression in the Intervention group

	IFTA progression - *N* = 33	IFTA progression + *N* = 19	*P*-value
Baseline characteristics			
Recipient age (years)	53 [40–63]	59 [52–62]	0.337
Recipient sex (male, %)	14 (42.4)	10 (52.6)	0.673
Dialysis duration (months)	126 [88–177]	128 [73–169]	0.879
Body mass index (kg/m^2^)	20.5 [18.6–21.6]	21.5 [18.9–22.5]	0.430
Diabetic kidney disease	6 (18.2)	1 (5.3)	0.372
Donor age (years)	57 [50–65]	60 [48–63]	0.871
Donor sex (male, %)	19 (57.6)	8 (42.1)	0.431
Donor type (deceased, %)	7 (21.2)	3 (15.8)	0.910
Preformed DSA (%)	0 (0.0)	0 (0.0)	NA
ABO blood type incompatible KTx (%)	11 (33.3)	10 (52.6)	0.284
IFTA at 1-h biopsy (%)	6 (18.2)	1 (5.3)	0.372
IFTA at 1-year biopsy (%)	6 (18.2)	19 (100.0)	< 0.001
Interval between KTx and intervention (months)	1 [0–7]	6 [3–9]	0.012
Lab data 1 year after KT			
Calcium (mg/dL)	9.9 [9.2–10.4]	9.6 [9.0–10.1]	0.387
Phosphorus (mg/dL)	3.3 [2.7–3.8]	3.4 [3.0–3.7]	0.947
Intact PTH (pg/mL)	76.3 [34.8–51.0]	48.0 [10.0–99.4]	0.035
Hemoglobin (g/dL)	12.0 [10.9–13.4]	12.1 [11.2–13.2]	0.909
LDL-C (mg/dL)	95.0 [84.0–112.0]	107.0 [99.5–122.5]	0.023
Uric acid (mg/dL)	6.4 [5.7–7.6]	7.3 [5.8–7.8]	0.430
eGFR (mL/min/1.73m^2^)	44.2 [37.8–51.0]	44.3 [29.4–48.5]	0.537
Urinary protein (g/g creatinine)	0.04 [0.00–0.12]	0.05 [0.00–0.14]	0.779
Tacrolimus trough level (ng/mL)	4.5 [4.2–5.8]	5.3 [4.2–5.7]	0.448
Cyclosporine trough level (ng/mL)	81.0 [68.0–101.0]	80.0 [72.0–112.0]	0.469
Mean blood pressure (mmHg)	86.7 [81.3–91.0]	89.7 [86.0–93.8]	0.100
BPR within 1 year after KTx (%)	0 (0.0)	1 (5.3)	0.778
Graft loss (%)	0 (0.0)	1 (5.3)	0.778

*BPR* *biopsy-proven rejection,* *DSA donor-specific human leukocyte antigen antibody,* *eGFR estimated glomerular filtration rate,* *IFTA interstitial fibrosis and tubular atrophy,* *LDL-C* low-density lipoprotein cholesterol, *NA* *not applicable,* *PTH parathyroid hormone*

**Fig. 4:**
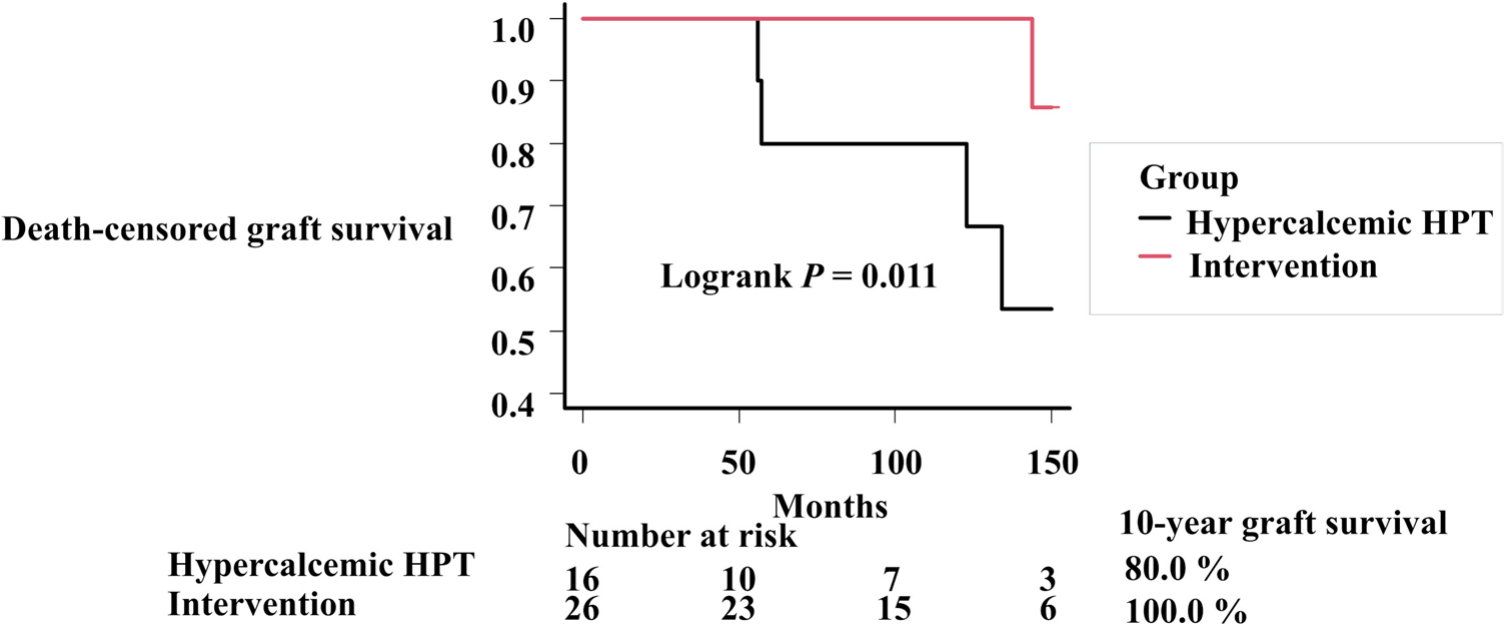
Death-censored graft survival curves in IFTA-positive cases in the normocalcemic HPT and intervention groups. *HPT* hyperparathyroidism, *IFTA* interstitial fibrosis and tubular atrophy

Among cases with IFTA at the 1-year graft biopsy, graft survival was significantly better in the intervention group than in the hypercalcemic HPT group, despite no significant differences in background characteristics between the two groups, except for dialysis duration, serum Ca, P, and PTH levels (Table 6 and Fig. 4).

**Table 6:** Patient backgrounds with IFTA at protocol biopsy 1 year post -KTx in the hypercalcemic HPT and Intervention groups

	Hypercalcemic HPT *N* = 16	Intervention *N* = 26	*P*-value
Baseline characteristics			
Recipient age (years)	57 [44–62]	60 [53–63]	0.510
Recipient sex (male, %)	11 (68.8)	15 (57.7)	0.697
Dialysis duration (months)	84 [23–120]	151 [75–180]	0.028
Body mass index (kg/m^2^)	22.7 [20.5–24.8]	21.5 [19.0–22.7]	0.095
Diabetic kidney disease	2 (12.5)	2 (7.7)	1.000
Donor age (years)	58 [54–65]	60 [48–64]	0.726
Donor sex (male, %)	6 (37.5)	13 (50.0)	0.638
Donor type (deceased, %)	4 (25.0)	8 (30.8)	0.960
Preformed DSA (%)	1 (6.2)	0 (0.0)	0.804
ABO blood type incompatible KTx (%)	5 (31.2)	11 (42.3)	0.697
Lab data 1 year after KT			
Calcium (mg/dL)	10.9 [10.8–11.2]	9.6 [9.2–10.1]	< 0.001
Phosphorus (mg/dL)	2.8 [2.6–2.9]	3.4 [2.9–3.7]	0.004
Intact PTH (pg/mL)	159.0 [131.3–211.3]	52.0 [12.5–121.0]	< 0.001
Hemoglobin (g/dL)	12.4 [11.5–13.0]	12.1 [11.1–13.1]	0.604
LDL-C (mg/dL)	97.5 [79.5–107.0]	105.0 [94.0–119.8]	0.100
Uric acid (mg/dL)	7.5 [6.0–7.6]	7.3 [6.0–7.8]	0.928
eGFR (mL/min/1.73m^2^)	41.1 [36.0–55.5]	40.1 [27.7–47.8]	0.170
Urinary protein (g/g creatinine)	0.02 [0.00–0.11]	0.03 [0.00–0.13]	0.610
Tacrolimus trough level (ng/mL)	5.1 [4.5–5.6]	5.3 [4.3–6.1]	0.664
Cyclosporine trough level (ng/mL)	94.0 [76.0–115.8]	78.0 [69.0–97.0]	0.568
Mean blood pressure (mmHg)	84.2 [77.6–90.8]	88.3 [83.5–92.3]	0.078
BPR within 1 year after KTx (%)	1 (6.2)	1 (3.8)	1.000
Graft loss (%)	5 (31.2)	1 (3.8)	0.044

*BPR* biopsy-proven rejection, *DSA* donor-specific human leukocyte antigen antibody, *eGFR* estimated glomerular filtration rate, *HPT* hyperparathyroidism, *IFTA* interstitial fibrosis and tubular atrophy, *LDL-C* low-density lipoprotein cholesterol, *PTH* parathyroid hormone

Eight patients had tubulointerstitial calcification detected at the 1-h protocol biopsy. Of these, six showed no calcifications at the 1-year biopsy, and two did not undergo it. New calcifications were detected at the 1-year biopsy in 48 patients, with a higher proportion in the intervention group (Table 3). Logistic regression revealed a significant association between mean PTH levels and calcification occurrence (Online Resource 5).

## Discussion

In this study, the risk of death-censored graft loss was significantly higher in the untreated hypercalcemic HPT group compared to the normal group. Contrarily, the risk of graft loss in the intervention group, which received treatment for hypercalcemic HPT within the first year after KTx, was comparable to that of the normal group. These trends persisted even after adjusting for multiple covariates in the multivariate analysis, suggesting that early treatment for hypercalcemic HPT may improve kidney graft survival. The findings may support informed decision-making in the management of post-transplantation HPT. In a retrospective study of 280 KTx patients with hypercalcemic HPT, Wang et al. demonstrated that treatment for hypercalcemic HPT was associated with improved kidney-graft survival [16]. However, to the best of our knowledge, no other studies have reported the association between interventions for HPT and kidney-graft survival.

IFTA is characterized by an excessive proliferation of extracellular matrix, which is the common chronic pathology of almost all persistent kidney injury [10]. Factors known to cause IFTA include rejection [17], metabolic syndrome [18, 19], infection [20], inflammation [21], ischemia/hypoxia [22–24], and CNI [25]. The severity of IFTA correlates with kidney graft function and survival [26, 27]. In this study, we examined protocol biopsies at 1-h and 1-year post-KTx to analyze the association between hypercalcemic HPT and IFTA progression. A high proportion of IFTA progression cases was observed in both the hypercalcemic HPT and intervention groups. In addition, multivariate logistic regression analysis revealed a significant association between hypercalcemia during the first year post-KTx and IFTA progression. In the intervention group, delayed treatment was likely to be associated with IFTA progression. Although few studies have directly linked hypercalcemia to IFTA, vascular smooth muscle contraction caused by persistent hypercalcemia [28] may lead to chronic ischemia in the kidney graft, potentially contributing to the progression of IFTA. These findings suggest that hypercalcemic HPT may drive the progression of IFTA in kidney grafts. On the other hand, among the IFTA-positive cases, graft survival was poor in the hypercalcemic HPT group, whereas the intervention group had good outcomes. While protocol biopsies were unavailable beyond 1 year after KTx, interventions correcting hypercalcemia might have suppressed further IFTA progression, improving outcomes. Considered together, early treatment for hypercalcemic HPT could be beneficial to prevent hypercalcemia-related IFTA progression, improving graft survival. We also demonstrated a potential link between tubulointerstitial calcification in kidney grafts and elevated PTH levels. While such calcification may impact graft prognosis, its low incidence in our cohort makes its overall effect unclear.

Although HPT after KTx should be treated appropriately, the potential increase in serum creatinine levels after PTx is an important issue [29, 30]. Some reports demonstrated an association between PTx within 1 year after KTx and elevated creatinine levels [29, 31]. Reports on PTx and increased serum creatinine levels are conflicting [32], but even a temporary increase would be of concern for transplant nephrologists and patients. To correct hypercalcemia early while avoiding creatinine elevation, it may be a good practice to introduce calcimimetics as initial treatment and perform PTx one year or more after KTx.

Both PTx and calcimimetics are effective treatment options for HPT after KTx; however, each has advantages and disadvantages. PTx offers a strong therapeutic effect but is invasive, whereas calcimimetics are non-invasive but costly [33] and may increase the risk of urinary stones [34]. In the only randomized controlled trial comparing PTx and cinacalcet in patients who underwent KTx, PTx was suggested to be superior to cinacalcet in improving bone loss and controlling hypercalcemia [35]. However, in terms of kidney graft survival, whether PTx or calcimimetics is superior as a treatment for hypercalcemic HPT is unclear. Therefore, the HPT treatment should be carefully selected based on each case, considering the patient’s kidney function, bone fragility, financial situation, and severity of hypercalcemia.

This study has some limitations, including those due to the study’s retrospective nature, confinement to a single center, lack of information on other contributing factors to the pathogenesis of IFTA, the potential presence of unmeasured confounders, selection bias, and the inability to establish a causal relationship between HPT management and kidney graft outcomes due to the retrospective design. Therefore, further randomized controlled trials with larger sample sizes are required to validate the findings of this study.

## Conclusion

Hypercalcemic HPT is associated with inferior kidney graft survival and IFTA progression. Proactive management of HPT may reduce the risk of graft loss and mitigate IFTA progression.

## Supplementary Information

Below is the link to the electronic supplementary material.

Supplementary file1 (DOCX 20 KB)

Supplementary file2 (DOCX 18 KB)

Supplementary file3 (DOCX 21 KB)

Supplementary file4 (DOCX 18 KB)

Supplementary file5 (DOCX 19 KB)

## Data Availability

The datasets generated and/or analyzed in the current study are available from the corresponding author upon reasonable request.
