# Supplementary material for: Post-transplantation management of hyperparathyroidism and its association with kidney graft survival and fibrosis
Source: Clin Exp Nephrol. 2025 Jul 4;29(12):1881–91. doi: 10.1007/s10157-025-02723-7 (PMC12660426; doi:10.1007/s10157-025-02723-7)
Supplement: Supplementary file 1 — Supplementary file1 (DOCX 20 KB) [file 10157_2025_2723_MOESM1_ESM.docx]

| **Table S1** Univariate Cox hazard regression for death-censored graft loss | | | |
| --- | --- | --- | --- |
|  | *P*-value | HR | 95% CI |
| Recipient age (years) | 0.998 | 1.00 | 0.99–1.02 |
| Male recipient | 0.043 | 1.57 | 1.01–2.44 |
| Dialysis duration (months) | 0.345 | 1.00 | 0.99–1.01 |
| Body mass index (kg/m^2^) | 0.007 | 1.07 | 1.02–1.13 |
| Diabetic kidney disease | 0.402 | 0.78 | 0.44–1.38 |
| Deceased donor | 0.108 | 1.88 | 0.87–4.06 |
| Donor age (years) | 0.035 | 1.02 | 1.00–1.04 |
| Male donor | 0.557 | 0.88 | 0.58–1.34 |
| Preformed DSA | 0.008 | 2.28 | 1.25–4.18 |
| ABO blood type incompatibility | 0.842 | 0.96 | 0.62–1.47 |
| Phosphorus (mg/dL) | 0.059 | 1.33 | 0.99–1.80 |
| Hemoglobin (g/dL) | <0.001 | 0.79 | 0.69–0.90 |
| LDL-C (mg/dL) | 0.309 | 0.99 | 0.99–1.01 |
| Uric acid (mg/dL) | <0.001 | 1.34 | 1.17–1.53 |
| eGFR (mL/min/1.73m2) | <0.001 | 0.94 | 0.92–0.96 |
| Proteinuria (reference to normal) |  | | |
| Mild | <0.001 | 2.50 | 1.57–3.98 |
| Severe | <0.001 | 6.54 | 3.88–11.03 |
| CNI trough level (reference to low) |  | | |
| Medium | 0.478 | 1.21 | 0.71–2.06 |
| High | 0.434 | 1.23 | 0.73–2.06 |
| Mean blood pressure (mmHg) | 0.288 | 1.01 | 0.99–1.04 |
| BPR within 1year after KTx | <0.001 | 4.76 | 2.91–7.79 |
| 95% CI*, 95% confidence interval;* BPR, *biopsy-proven rejection;* CNI, *calcineurin inhibitor;* DSA*, donor-specific human leukocyte antigen antibody;* eGFR*, estimated glomerular filtration rate;* HPT*, hyperparathyroidism;* HR*, hazard ratio;* KTx*, kidney transplantation;* LDL-C*, low-density lipoprotein cholesterol.* | | | |
