# Supplementary material for: Post-transplantation management of hyperparathyroidism and its association with kidney graft survival and fibrosis
Source: Clin Exp Nephrol. 2025 Jul 4;29(12):1881–91. doi: 10.1007/s10157-025-02723-7 (PMC12660426; doi:10.1007/s10157-025-02723-7)
Supplement: Supplementary file 2 — Supplementary file2 (DOCX 18 KB) [file 10157_2025_2723_MOESM2_ESM.docx]

| **Table S2. Variance inflation factor (VIF) in the multivariate Cox hazard model** | | | |
| --- | --- | --- | --- |
| **Variables** | **Generalized VIF** | **Degrees of freedom (Df)** | **Generalized VIF^(1/(2*Df))** |
| HPT management status | 1.715455 | 3 | 1.0941 |
| Recipient sex | 1.338206 | 1 | 1.1568 |
| Recipient age | 1.09712 | 1 | 1.0474 |
| eGFR at 1 year post KTx | 1.596367 | 1 | 1.2635 |
| Proteinuria | 1.253895 | 2 | 1.0582 |
| Body mass index | 1.172029 | 1 | 1.0826 |
| Dialysis duration | 2.170276 | 1 | 1.4732 |
| Donor age | 1.323183 | 1 | 1.1503 |
| Donor type | 1.719643 | 1 | 1.3114 |
| Biopsy-proven rejection within 1year after KTx | 1.156999 | 1 | 1.0756 |
| Preformed　DSA | 1.241939 | 1 | 1.1144 |
| Serum phosphorus | 1.310973 | 1 | 1.145 |
| Hemoglobin | 1.455063 | 1 | 1.2063 |
| Uric acid | 1.217671 | 1 | 1.1035 |
| Mean blood pressure | 1.142974 | 1 | 1.0691 |
