# Supplementary material for: Post-transplantation management of hyperparathyroidism and its association with kidney graft survival and fibrosis
Source: Clin Exp Nephrol. 2025 Jul 4;29(12):1881–91. doi: 10.1007/s10157-025-02723-7 (PMC12660426; doi:10.1007/s10157-025-02723-7)
Supplement: Supplementary file 3 — Supplementary file3 (DOCX 21 KB) [file 10157_2025_2723_MOESM3_ESM.docx]

| **Table S3** Univariate logistic hazard regression for IFTA progression | | | |
| --- | --- | --- | --- |
|  | *P*-value | OR | 95% CI |
| Recipient age (years) | 0.337 | 0.99 | 0.99–1.01 |
| Male recipient | 0.209 | 0.84 | 0.64–1.10 |
| Dialysis duration (months) | 0.041 | 1.00 | 1.00–1.01 |
| Body mass index (kg/m^2^) | 0.119 | 0.97 | 0.93–1.01 |
| Diabetic kidney disease | 0.953 | 0.99 | 0.70–1.40 |
| Deceased donor | 0.116 | 1.63 | 0.89–2.99 |
| Donor age (years) | 0.005 | 1.02 | 1.01–1.03 |
| Male donor | 0.321 | 1.15 | 0.87–1.51 |
| Preformed DSA | 0.275 | 0.72 | 0.40–1.30 |
| Phosphorus (mg/dL) | 0.367 | 1.14 | 0.86–1.52 |
| Intact PTH (pg/mL) | 0.589 | 1.00 | 0.99–1.00 |
| Hemoglobin (g/dL) | <0.001 | 0.78 | 0.70–0.87 |
| LDL-C (mg/dL) | 0.74 | 1.00 | 0.99–1.01 |
| Uric acid (mg/dL) | 0.002 | 1.19 | 1.07–1.33 |
| eGFR (mL/min/1.73m2) | <0.001 | 0.97 | 0.96–0.98 |
| Proteinuria (reference to normal) |  | | |
| Mild | 0.136 | 1.29 | 0.92–1.82 |
| Severe | 0.008 | 2.05 | 1.21–3.46 |
| CNI trough level (reference to low) |  | | |
| Medium | 0.808 | 1.04 | 0.75–1.46 |
| High | 0.285 | 1.20 | 0.86–1.66 |
| Mean blood pressure (mmHg) | 0.153 | 0.99 | 0.97–1.00 |
| BPR within 1year after KTx | 0.018 | 1.93 | 1.12–3.31 |
| The average values of serum phosphorus, hemoglobin, LDL-C, uric acid, intact PTH, and CNI levels during the first year after KTx were used.  95% CI*, 95% confidence interval;* BPR, *biopsy-proven rejection;* CNI, *calcineurin inhibitor;* DSA*, donor-specific human leukocyte antigen antibody;* eGFR*, estimated glomerular filtration rate;* HPT*, hyperparathyroidism;* IFTA*, interstitial fibrosis and tubular atrophy;* KTx*, kidney transplantation;* LDL-C*, low-density lipoprotein cholesterol;* OR*, odds ratio;* PTH, *parathyroid hormone.* | | | |
