# Supplementary material for: Post-transplantation management of hyperparathyroidism and its association with kidney graft survival and fibrosis
Source: Clin Exp Nephrol. 2025 Jul 4;29(12):1881–91. doi: 10.1007/s10157-025-02723-7 (PMC12660426; doi:10.1007/s10157-025-02723-7)
Supplement: Supplementary file 4 — Supplementary file4 (DOCX 18 KB) [file 10157_2025_2723_MOESM4_ESM.docx]

| **Table S4 Variance inflation factor (VIF) in the logistic regression** | | | |
| --- | --- | --- | --- |
| **Variables** | **Generalized VIF** | **Degrees of freedom (Df)** | **Generalized VIF^(1/(2*Df))** |
| Recipient age | 1.238482 | 1 | 1.112871 |
| Recipient sex | 1.551142 | 1 | 1.245448 |
| eGFR | 1.634309 | 1 | 1.278401 |
| Proteinuria | 1.148201 | 2 | 1.035153 |
| Donor age | 1.51705 | 1 | 1.231686 |
| Donor type | 1.512433 | 1 | 1.22981 |
| Biopsy-proven rejection within 1year after KTx | 1.081123 | 1 | 1.03977 |
| Preformed DSA | 1.088188 | 1 | 1.043162 |
| Body mass index | 1.296732 | 1 | 1.138741 |
| Hemoglobin | 1.465737 | 1 | 1.210676 |
| Diabetic kidney disease | 1.23952 | 1 | 1.113337 |
| low-density lipoprotein cholesterol | 1.082012 | 1 | 1.040198 |
| Uric acid | 1.511788 | 1 | 1.229548 |
| Calcineurin inhibitor level | 1.108834 | 2 | 1.026164 |
| Intact PTH | 1.232522 | 1 | 1.11019 |
| Mean blood pressure | 1.104353 | 1 | 1.050882 |
| Dialysis duration | 1.936165 | 1 | 1.391461 |
| Serum phosphorus | 1.358999 | 1 | 1.165761 |
| Serum calcium | 1.566056 | 1 | 1.251422 |
