# Supplementary material for: Post-transplantation management of hyperparathyroidism and its association with kidney graft survival and fibrosis
Source: Clin Exp Nephrol. 2025 Jul 4;29(12):1881–91. doi: 10.1007/s10157-025-02723-7 (PMC12660426; doi:10.1007/s10157-025-02723-7)
Supplement: Supplementary file 5 — Supplementary file5 (DOCX 19 KB) [file 10157_2025_2723_MOESM5_ESM.docx]

| **Table S5** Logistic regression analysis of tubulointerstitial calcification in the 1-year protocol biopsy post-KTx. | | | |
| --- | --- | --- | --- |
|  | Factors | OR [95% CI] | *P-*value |
| Model 1 | Serum calcium (mg/dL) | 2.22 [1.29–3.82] | 0.004 |
| Model 2 | Serum calcium (mg/dL) | 1.90 [1.11–3.28] | 0.020 |
|  | Intact PTH (pg/mL) | 1.00 [1.00–1.01] | <0.001 |
| Model 3 | Serum calcium (mg/dL) | 1.66 [0.93–2.97] | 0.088 |
|  | Intact PTH (pg/mL) | 1.00 [1.00–1.01] | 0.004 |
|  | Serum phosphorus (mg/dL) | 0.66 [0.33–1.31] | 0.233 |
| Model 4 | Serum calcium (mg/dL) | 1.56 [0.86–2.83] | 0.144 |
|  | Intact PTH (pg/mL) | 1.00 [1.00–1.01] | 0.005 |
|  | Serum phosphorus (mg/dL) | 0.63 [0.32–1.26] | 0.195 |
|  | CNI trough level (reference to low) |  | |
|  | Medium | 1.17 [0.52–2.61] | 0.703 |
|  | High | 1.78 [0.84–3.77] | 0.135 |
| Serum calcium, intact PTH, phosphorus, and CNI levels were included as potential contributors, and their average values over the first year after KTx were used in the analysis. CNI trough levels were categorized into three tertiles: low, medium, and high.  95% CI*, 95% confidence interval;* CNI, *calcineurin inhibitor;* KTx*, kidney transplantation;* OR*, odds ratio;* PTH, *parathyroid hormone* | | | |
